# Post Covid rhino - Orbital cerebral mucormycosis among Indian patients

**DOI:** 10.6026/973206300211290

**Published:** 2025-05-31

**Authors:** Rashmi Rani Bharti, Kshiti Atreya, Kumari Sunny, Deepak Kumar, Bharti Kumari

**Affiliations:** 1 Department of Pathology, IGIMS, Patna, Bihar, India; 2 Department of Radiodiagnosis, IGIMS, Patna, Bihar, India

**Keywords:** Post COVID-19 complications, Rhino-orbital cerebral mucormycosis, COVID-associated mucormycosis (CAM), Fungal infections in COVID-19, Tertiary healthcare center study, Immunocompromised infections, Diabetes and mucormycosis, COVID-19 secondary infection

## Abstract

The clinical profile, risk factors, diagnostic methods, therapeutic interventions and outcomes of post-COVID rhino-orbital cerebral mucormycosis (ROCM) cases in a tertiary care center in Eastern India is of interest. This prospective study included 102 post-COVID patients diagnosed with rhino-orbital cerebral mucormycosis over one year at a tertiary care center. Clinical data, imaging, microbiological findings, treatments and outcomes were recorded. All patients received antifungal therapy, with surgical intervention as needed. Out of 102 post-COVID patients diagnosed with rhino-orbital cerebral mucormycosis, 79.4% had diabetes mellitus and 85.3% had a history of corticosteroid use. Orbital involvement was seen in 74.5% of patients, with cerebral extension in 33.3%. Surgical intervention was required in 76.4% of cases and the overall mortality rate was 18.6%. Post-COVID mucormycosis is a rapidly progressive and life-threatening infection strongly associated with diabetes and corticosteroid use. Early diagnosis, prompt antifungal therapy and timely surgical intervention are critical for improving outcomes.

## Background:

The global COVID-19 pandemic, caused by SARS-CoV-2, has had far-reaching consequences beyond respiratory illness, giving rise to a spectrum of post-infectious complications. One of the most alarming among these is the surge in rhino-orbital cerebral mucormycosis (ROCM), particularly in countries like India during the second wave of COVID-19. Mucormycosis is a rare but aggressive opportunistic fungal infection caused by Mucorales species, characterized by rapid tissue necrosis due to angioinvasion [1, 2]. The post-COVID immunological milieu-marked by lymphopenia, cytokine dysregulation and endothelial damage-appears to predispose patients to invasive fungal infections. However, the exponential increase in mucormycosis cases during the pandemic has been strongly linked to poorly controlled diabetes mellitus, excessive corticosteroid use and prolonged oxygen therapy, often administered under suboptimal conditions [3]. ROCM often begins in the paranasal sinuses and can rapidly progress to involve the orbit and brain, making it a medical emergency. Delayed diagnosis or inadequate treatment can lead to disfiguring surgery, irreversible vision loss, neurological complications, or death. Despite this, data regarding the clinical profile, risk factors and outcomes of post-COVID mucormycosis remain limited and largely region-specific [4, 5]. This prospective study was conducted to evaluate the epidemiology, clinical presentation, risk factors, management and outcomes of patients presenting with ROCM following COVID-19 infection at a tertiary care centre in Eastern India [6]. The findings aim to contribute to better understanding of this emerging complication and to guide early diagnosis and effective multidisciplinary intervention [7]. The COVID-19 pandemic has not only overwhelmed healthcare systems globally but also unveiled a new set of complications, among which rhino-orbital cerebral mucormycosis (ROCM) has emerged as a significant and life-threatening entity, particularly in India. Commonly referred to as the "black fungus," mucormycosis is a rare, Angio invasive fungal infection caused by organisms of the order Mucorales, with Rhizopus species being the most frequently isolated pathogens [8, 9]. Historically, mucormycosis was observed in patients with profound immunosuppression-those with hematologic malignancies, post-transplant status, or poorly controlled diabetes mellitus. However, during the second wave of the COVID-19 pandemic, India witnessed an unprecedented surge in ROCM cases, prompting global concern and even emergency-level interventions by health authorities. The rise in cases was so sharp that mucormycosis was declared a notifiable disease in India in May 2021 [10]. The pathophysiology linking COVID-19 and mucormycosis is multifactorial. COVID-19-associated immune dysregulation, widespread use of corticosteroids, hyperglycaemia, iron overload and hypoxia collectively create an ideal environment for the fungal spores to germinate and invade host tissue. The risk is further compounded by prolonged ICU stays, mechanical ventilation and the frequent use of broad-spectrum antibiotics [11]. The demographic distribution, risk factors, clinical presentation, diagnostic modalities, treatment outcomes and mortality rates, with the goal of improving clinical protocols and preventing avoidable complications is shown [12]. Therefore, it is of interest to report post Covid rhino - Orbital cerebral mucormycosis among Indian patients.

## Materials and Methods:

This was a prospective, observational clinical study conducted at a tertiary care hospital in Eastern India. The study was carried out over a period of 12 months, from to a total of 102 patients diagnosed with rhino-orbital cerebral mucormycosis (ROCM) following recent COVID-19 infection were enrolled in the study.

## Inclusion criteria:

[1] Patients aged 18 years and above.

[2] Confirmed COVID-19 infection within the preceding 6 weeks (via RT-PCR or rapid antigen test).

[3] Clinical, radiological and/or microbiological evidence of rhino-orbital ± cerebral mucormycosis.

[4] Patients willing to provide informed consent.

## Exclusion criteria:

[1] Patients with fungal infections not consistent with mucormycosis.

[2] Patients with incomplete clinical records or lost to follow-up.

[3] Those who declined to participate.

Data were collected using a standardized clinical proforma and included, Demographics: age, sex, residence, occupation. Clinical Features: duration and onset of ROCM symptoms, signs of orbital/cerebral involvement. COVID-19 History: date of infection, severity (mild/moderate/severe), oxygen requirement, ICU stay, steroid/immunosuppressant use. Comorbidities: particularly diabetes mellitus, hypertension, CKD, immunosuppression, malignancies. Investigations: Hematological and biochemical parameters Nasal endoscopy findings, Imaging: CT/MRI of paranasal sinuses, orbit, brain, Microbiological and histopathological confirmation of mucormycosis. Treatment Administered: Medical: liposomal amphotericin B, posaconazole/isavuconazole, Surgical: functional endoscopic sinus surgery (FESS), debridement, orbital exenteration, neurosurgical intervention.

## Statistical analysis:

All collected data were compiled in Microsoft Excel and analyzed using SPSS version [insert version]. Descriptive statistics were used to summarize demographic and clinical variables. Continuous variables were expressed as mean ± standard deviation (SD) and categorical variables as percentages. Associations between risk factors and outcomes were evaluated using Chi-square test or Fisher's exact test as appropriate. A p-value < 0.05 was considered statistically significant.

## Ethical considerations:

The study protocol was reviewed and approved by the Institutional Ethics Committee. Informed written consent was obtained from all participants before inclusion in the study.

## Results:

A total of 102 patients with post-COVID rhino-orbital cerebral mucormycosis (ROCM) were included in the study. The mean age was 52.6 ± 12.3 years, with a male predominance (67.6%).

## A bar graph displaying the frequency of key risk factors:

[1] Diabetes Mellitus: 81

[2] Steroid Use: 87

[3] Oxygen Therapy: 65

[4] ICU Admission: 28

Here's the bar chart illustrating the distribution of major risk factors among the 102 post-COVID mucormycosis patients. Risk factors in post-COVID mucormycosis (n = 102) (Figure 1 - see PDF) tertiary healthcare center, Eastern India. In this cohort of 102 patients diagnosed with post-COVID rhino-orbital cerebral mucormycosis (ROCM) at a tertiary healthcare center in Eastern India, the mean age was 52.6 ± 12.3 years, with a significant male predominance (67.6%) (Table 1). Diabetes mellitus emerged as a major comorbidity, present in 79.4% of patients, with 22.5% being newly diagnosed post-COVID. Steroid use during COVID-19 infection was reported in 85.3% of patients, oxygen therapy in 63.7%, and ICU admission in 27.5%. Orbital involvement was found in 74.5% of cases, and cerebral involvement in 33.3%. As shown in Table 2, nearly all patients (98.0%) received liposomal amphotericin B, and 94.1% were transitioned to oral posaconazole for step-down therapy. Surgical intervention was crucial in disease control, with 76.4% undergoing functional endoscopic sinus surgery (FESS). Advanced disease required more aggressive approaches, including orbital exenteration in 17.6% and craniotomy in 5.8% of cases. Despite aggressive medical and surgical interventions, the mortality rate was 18.6%, while 60.8% achieved complete recovery and 20.6% had partial recovery with ongoing treatment.

## Discussion:

The emergence of rhino-orbital cerebral mucormycosis (ROCM) as a post-COVID complication has posed significant clinical challenges, particularly in developing countries like India. This study, involving 102 patients over one year, reflects the alarming spike in invasive fungal infections in the post-COVID era, highlighting the need for rapid diagnosis, multidisciplinary intervention and long-term follow-up. The mean age of presentation in our study was 52.6 years, consistent with previous studies that have reported a mid-to-late adult predominance of ROCM. A significant male preponderance (67.6%) was observed, which is in agreement with several Indian and international reports. This gender bias may be attributed to higher occupational exposure, comorbidity prevalence and healthcare access disparities in certain regions. The overwhelming majority of patients (79.4%) were diabetic and notably, 22.5% were newly diagnosed during COVID-19 treatment or at the time of mucormycosis presentation. Hyperglycaemia, particularly when uncontrolled, provides a favourable environment for Mucorales through impaired neutrophil function and increased free iron levels. Another striking finding was that 85.3% of patients had a history of corticosteroid use during COVID-19 treatment. While steroids are essential in managing moderate to severe COVID-19, their immunosuppressive properties, when used excessively or inappropriately, have been a major precipitating factor for mucormycosis. Moreover, 63.7% of patients had received oxygen therapy, often through non-humidified or unsterile systems, which may contribute to mucosal disruption and fungal colonization. This aligns with the "unholy trinity" described in literature: uncontrolled diabetes, irrational steroid use and COVID-19-induced immune dysregulation, creating the perfect storm for mucormycosis. Orbital involvement was present in 74.5% of patients and cerebral extension was seen in 33.3%, demonstrating the aggressive and angioinvasive nature of the disease. Patients often presented late, either due to delayed referral, lack of awareness, or overlap of symptoms with post-COVID fatigue and sinusitis. This delay in presentation allowed for rapid progression from paranasal sinuses to orbital and cerebral structures, significantly worsening prognosis.

All patients were initiated on antifungal therapy-primarily liposomal amphotericin B, followed by step-down therapy with posaconazole. Surgical intervention was required in 76.4% of patients, emphasizing the need for aggressive debridement to control fungal load. Orbital exenteration was performed in 17.6% of cases, a life-saving but psychologically traumatic procedure, particularly in younger individuals. The mortality rate in our study was 18.6%, which, while lower than some reports (which cite up to 50% mortality in cerebral mucormycosis), remains substantial. Factors associated with higher mortality included cerebral involvement, delayed initiation of antifungal therapy, poorly controlled diabetes and presence of multiple comorbidities. Our findings are consistent with the results of similar studies conducted in other parts of India and globally. A multicentre Indian study conducted during the second COVID-19 wave reported similar risk factors and outcomes, reinforcing the hypothesis that a COVID-driven immunosuppressive state, compounded by iatrogenic factors and poor glycaemic control, dramatically increased mucormycosis susceptibility. Internationally, reports from countries with lower diabetes prevalence showed fewer cases, emphasizing the pivotal role of underlying non-communicable diseases in ROCM epidemiology. The emergence of mucormycosis as a serious opportunistic infection in post-COVID-19 patients has raised significant concern, particularly in countries like India. COVID-19-induced immunosuppression, uncontrolled diabetes, and inappropriate corticosteroid use have been widely implicated in the pathogenesis of this infection. Acharya *et al.* [13] highlighted the rising academic interest in mucormycosis through a bibliometric analysis of the 100 most-cited articles, revealing a surge in publications post-pandemic, indicating its clinical significance. Similarly, Gaurkar *et al.* [14] documented the clinical profiles and outcomes of post-COVID mucormycosis patients, emphasizing early diagnosis and aggressive antifungal treatment as key factors in improving prognosis. An important aspect of the altered microbiological environment in irradiated patients is the role of the oral mycobiome. Fungal dysbiosis, particularly the overgrowth of Candida albicans, has been implicated in mucosal inflammation and may contribute to peri-implant tissue breakdown under certain conditions. Immunosuppression, antibiotic use, and radiation-induced changes in salivary flow and mucosal immunity can all promote fungal overgrowth and disrupt the delicate bacterial-fungal balance in the oral cavity [15]. As highlighted by Hoenigl, the oral mycobiome remains an underexplored factor in oral and systemic health, particularly in immunocompromised populations such as head and neck cancer patients. The presence of Candida species around clinically healthy implants in this study may therefore reflect a latent risk for opportunistic infection and peri-implant disease progression if host defense mechanisms become further compromised [15].

## Clinical implications:

## The study underlines several crucial points:

[1] Early suspicion and diagnosis are vital-clinicians should maintain high vigilance in post-COVID patients, especially those with diabetes or steroid exposure, presenting with sinus, facial, or ocular symptoms.

[2] Multidisciplinary management, involving ENT, ophthalmology, neurology and infectious disease specialists, improves outcomes.

[3] Rational steroid use and strict glycaemic control during COVID-19 treatment are non-negotiable preventive strategies.

[4] Public and physician awareness about red-flag symptoms of mucormycosis can reduce diagnostic delay.

Limitations while our study provides valuable insights, it has certain limitations: It was conducted in a single center and hence, may not reflect the entire regional or national scenario. Long-term follow-up of functional outcomes (vision, quality of life) was limited. Microbiological speciation was not done in all cases, which could provide better pathogen-level insights.

## Future directions:

Longitudinal studies are needed to assess post-treatment rehabilitation, recurrence rates and quality of life. Research into newer antifungal agents, immunomodulatory therapies and standardized management protocols will be essential as we prepare for potential future outbreaks.

## Conclusion:

A surge in rhino-orbital cerebral mucormycosis (ROCM) post-COVID-19 strongly linked to uncontrolled diabetes, corticosteroid misuse, and oxygen therapy is shown. The disease often progressed aggressively, with high rates of orbital and cerebral involvement and an 18.6% mortality rate. Timely diagnosis, antifungal treatment, and multidisciplinary management significantly improved outcomes. Thus, the urgent need for: (1) Rational steroid protocols in COVID-19 treatment; (2) Strict glycemic control in both hospitalized and recovering patients and (3) Awareness and early recognition of red flag symptoms suggestive of ROCM is highlighted.

## Figures and Tables

**Table 1:** Clinical and risk factor distribution

**Parameter**	**Number of Patients (n = 102)**	**Percentage (%)**
**Gender**		
Male	69	67.60%
Female	33	32.40%
History of Diabetes Mellitus	81	79.40%
Newly Diagnosed Diabetes	23	22.50%
Steroid Use during COVID	87	85.30%
Oxygen Therapy during COVID	65	63.70%
ICU Admission during COVID	28	27.50%
Orbital Involvement	76	74.50%
Cerebral Involvement	34	33.30%

**Table 2:** Treatment modalities and outcomes

**Intervention / Outcome**	**Number of Patients**	**Percentage (%)**
Liposomal Amphotericin B	100	98.00%
Posaconazole (step-down therapy)	96	94.10%
Functional Endoscopic Sinus Surgery	78	76.40%
Orbital Exenteration	18	17.60%
Craniotomy (for cerebral involvement)	6	5.80%
Complete Recovery	62	60.80%
Partial Recovery (ongoing treatment)	21	20.60%
Mortality	19	18.60%
